# Does project-based STEAM curriculum benefit 3–4-year-old preschoolers’ creative potential? A pilot comparison with theme-based curriculum

**DOI:** 10.3389/fpsyg.2026.1831871

**Published:** 2026-06-16

**Authors:** Duhong Peng

**Affiliations:** School of Education, Suzhou University of Science and Technology, Suzhou, China

**Keywords:** 3–4-year-old preschooler, creative potential, impact, project-based STEAM curriculum, traditional theme-based curriculum

## Abstract

Despite the increasing attention given to project-based STEAM education in early childhood settings, empirical evidence regarding its effects on the creative potential of very young children remains limited, particularly in comparison with traditional theme-based curricula. This pilot study employed a 1-year quasi-experimental design to examine the differential impacts of these two curricular approaches on the creative potential of 3–4-year-old preschool children. Sixty-nine children from three first-year preschool classes participated in the study, with individual assessments conducted before and after the intervention. The results indicated that, although the experimental group initially scored lower than the control group, it demonstrated greater improvement in overall creative potential over time. Within-group analyses revealed significant or marginally significant increases in the experimental group across multiple dimensions, including flexibility, fluency, and originality. In contrast, the control group exhibited no significant improvement and even exhibited a slight declining trend. Repeated-measures analyses further showed a significant Group × Time interaction for overall creative potential, indicating greater developmental gains in the experimental group. After controlling for baseline differences, the experimental group consistently demonstrated higher adjusted post-test means across all dimensions. Although these between-group differences did not reach statistical significance, moderate effect sizes suggested meaningful practical advantages. In addition, both groups exhibited increasing individual differences over time, with greater variability observed in the experimental group. Overall, the findings suggest that project-based STEAM curricula may offer advantages in promoting and sustaining early creative development compared with traditional theme-based approaches. This study provides preliminary empirical support for the implementation of project-based STEAM learning in early childhood education and highlights its potential role in fostering the development of creative potential during the early years.

## 1 Introduction

The recent advent of generative artificial intelligence (AI) is already greatly impacting not only what people can do but also what and how they think (Manalo et al., 2026). As humanity enters the era of AI, the last possible advantage of human intelligence is being challenged, and the ability to imagine and generate original ideas and products becomes crucial (Peng et al., 2026).

Creative potential is a unique potential ability or resource of an individual to generate original and effective ideas or solutions. It belongs to a latent state that constitutes part of an individual’s “human capital,” and creativity is the actual embodiment of this potential in the form of creative behavior, activities, or achievements (Agnoli et al., 2022; Barbot et al., 2015; Barbot et al., 2015). As creative potential provides a more appropriate construct for capturing children’s creative abilities (Runco, 2003), growing attention has been directed to school-age children’s creative potential in recent years (e.g., Cheung et al., 2016; Díaz-Pereira et al., 2025; Doz et al., 2026; Juriševič and Žerak, 2024; Khotinets and Shishova, 2023). Little is known about how creative potential develops in the early years, especially during the first year of preschool. Clarity on changes in creative potential in the very early years might help to complete the puzzle of the developmental laws of children’s creative potential during childhood and bring educational inspiration in the future.

### 1.1 Early childhood STEAM education and project-based STEAM inquiry

Despite extensive scholarly work on creativity promotion methods over two decades, a persistent challenge remains: translating creativity-promoting policies into effective classroom practices (Konstantinidou, 2026). STEAM education, as an interdisciplinary educational concept that directly targets complex real-world problems, has been incorporated into many developed countries’ national education development strategies, with a view to nurturing innovative and interdisciplinary applied talents for the twenty-first century and enhancing their core national competitiveness (Peng, 2024). Preschoolers are full of curiosity about the world, with countless questions in their minds and rich imagination and creativity; they have an innate enthusiasm for STEAM learning (DeJarnette, 2018).

According to Piaget’s cognitive development and constructivist theory (Glasersfield, 1995), children in early childhood are in the preoperational stage, characterized by concrete thinking, active exploration of the surrounding environment, and learning through direct sensory experience and hands-on manipulation. STEAM education aims at real-life problems that involve vivid phenomena, encouraging and engaging children to feel these authentic problems through hands-on and experiential activities. This completely matches the cognitive development characteristics of young children, enabling them to actively construct an understanding of the world rather than passively accept abstract concepts. Therefore, previous research and practice that took primary and secondary school STEAM education as the absolute mainstay have gradually expanded into the early years. Examples include *Baby Steps to STEM: Infant and Toddler* (Jean and Barbre, 2017). The extension of STEAM education to the early years has become a major international trend (Yang and Yang, 2021).

In promoting the quality of STEAM education, various implementation strategies have emerged. Project-based STEAM inquiry, which emphasizes real-world contexts and real problem-solving (Peng, 2024), is becoming a widely applied and prominent educational methodology (García-Llamas et al., 2025. It integrates the core commonalities of project-based inquiry and STEAM education. Through exploring challenging real-world problems, it achieves organic interdisciplinary integration and effective problem-solving.

Project-based inquiry is a continuous, in-depth process of overall investigation or research that is child-centered, follows children’s interests, and revolves around questions, rather than simply presenting knowledge related to a certain theme (Sargent, 2011). For example, why do humans love cats so much? If children have an interest, teachers can follow their interest to explore it as a project just focusing on one discipline. The uniqueness of STEAM projects lies in their formation in response to realistic, unsolved real-world problems, which integrate multiple disciplines and have core scientific and engineering problems behind them. For example, how can shoes be made non-slip in wet weather? Or given the severe global plastic waste pollution, can we create an environmentally friendly alternative to plastic? These real problems are inherently interdisciplinary, and the process of addressing complex real-world issues naturally integrates interdisciplinary practices. Therefore, STEAM projects do not need to overemphasize disciplinary boundaries; instead, they should focus on specific problems (Morrison, 2006). It directs students to concentrate on problem-solving rather than disciplinary content knowledge; it requires inquiry grounded in authentic projects (Wu, 2022). Therefore, STEAM project inquiry is highly expected to achieve high quality and bring about effective learning outcomes.

Studies have begun to examine the impact of a STEAM project-based learning (PBL) from higher education to secondary and primary education. For example, García-Llamas et al. (2025) investigated higher education students and academics from six universities, and the results indicated that both students and academics perceived similar benefits from PBL. For example, they reported high satisfaction and significant competency acquisition across all areas, regardless of their background or study modality. Rostikawati et al. (2024) demonstrated through experimental research that the academic performance of primary school students in the STEAM project learning experimental group improved by 77%, with a statistically significant difference between the pre-test and post-test compared to the control group utilizing conventional teaching methods. A quasi-experimental study indicated that project-based STEAM learning surpassed traditional instruction in cognitive learning outcomes at the elementary school level (Degeng et al., 2021). Nuraini et al. (2023) demonstrated an effective learning achievement rate of 88.57% in scientific literacy via STEAM PBL. Moreover, STEAM project-based inquiry can enhance students’ problem-solving and critical-thinking abilities (Carrillo et al., 2024) and substantially foster the advancement of creative thinking (Annisa et al., 2019; Pramashela et al., 2023). The findings indicate that STEAM projects enhance students’ higher-order thinking skills and academic achievement.

### 1.2 Creative potential in early childhood and traditional curriculums in preschool

Creative capacities are multifaceted; they may manifest themselves in verbal, movement, visual, or other forms in early childhood. The study of creative potential in kindergarten children is of vital importance (Fung and Chung, 2022). Studies have indicated that the creativity development of children in different types of kindergarten is generally low, and the score in the novelty dimension is particularly low (Peng et al., 2018). There are significant individual differences and early gaps in the development of creativity among second- and third-year preschoolers, and these individual differences tend to expand with age, with the individual differences of senior class children being significantly greater than those of second-year preschoolers (Peng et al., 2019). However, the quality of support provided by daily educational activities and environment creation in kindergartens for young children’s creativity development is relatively low (Peng and Liu, 2019).

Considering the advent and fast development of AI, creative potential is a significant personal asset for future civilization and is inherently found in every child (Belenkova et al., 2019). It is a pressing necessity to build a high-quality educational framework. Preschool education (including its curriculum and environment) requires optimization, innovation, transformation, and enhancement to effectively support the development of young children’s creative capacity, even though it is a challenging and urgent task. For example, traditional theme-based curricula in preschool classrooms are usually teacher-led (e.g., every cohort of children is offered a fixed set of thematic curricula each year), knowledge-oriented, and focused on superficial questions, with only a cursory, surface-level understanding.

Project-based STEAM methodology provides vivid and active classroom learning, makes children feel happy, and can enable them to express all the ideas in their imagination, so that children can create meaningful works (Habibi, 2023). Then what is its effect when it is implemented in early childhood classrooms? Theoretically, it can promote the development of children’s creative potential, as children’s imagination and curiosity reach their peak at this period. It is the most powerful time to form creative ideas and thoughts (Dilshodbekovna, 2023; Mukhametshin et al., 2020; Yıldırım and Yilmaz, 2023). However, there are few empirical studies exploring the impact of STEAM PBL on the creative potential of very young children (Atikah and Biru, 2024; Johnston et al., 2022; Zahra et al., 2023) existing research predominantly focuses on primary and secondary education (Rafiq-uz-Zaman et al., 2025; Sun et al., 2025).

It might be due to challenges in long-term interventions, difficulties in measuring young children’s creativity or creative potential, the complexity of interdisciplinary project design, or practical constraints such as the dominance of thematic or traditional approaches in early childhood settings. Consequently, only one study has emerged confirming the positive impact of STEAM PBL on the creative thinking of 4–6-year-old children with 30 participants (Zahra et al., 2023), which was also conducted without a comparative perspective. Much more empirical research is needed to verify the impacts of this novel model on children’s creative potential, including in different culture contexts and among very young children (e.g., the first-year preschool children).

### 1.3 The present study

Based on Piaget’s constructivist theory (Glasersfield, 1995) and phenomenon-based learning theory (Yu and Cao, 2020), the present study aims to enrich this field by exploring the impact of a purposefully designed project-based STEAM curriculum on the creative potential of 3–4-year-old preschool children. To test this more comprehensively, it also aimed to explore whether the STEAM project-based curriculum, which is characterized by greater openness, practicality, and in-depth integration compared with traditional thematic curricula, exerts a more significant enhancing effect on preschool children’s creative potential.

Unlike small-scale or case studies, this study will implement a structured intervention program, adopt a pre-and post-test design with an experimental group and a control group, and combine the quantitative measurement of creative potential to explore the impact of the STEAM project curriculum on the creative potential of 3–4-year-olds. This study will try to overcome practical challenges in long-term implementation, difficulties in assessment, and the complexity of STEAM project design, striving to explore whether the STEAM curriculum can improve creative potential and attempting to determine how to improve it by examining its specific effects on key dimensions.

The research results are expected to provide solid empirical evidence and practical inspiration for early childhood educators, helping them use STEAM PBL as a powerful, evidence-based strategy to cultivate a new generation of innovative thinkers. It is expected to not only deepen understanding of the relationship between curriculum models and the development of preschoolers’ higher-order thinking but also provide empirical evidence for the design and implementation of effective STEAM project programs that stimulate children’s innovative competencies in educational practice.

In summary, this pilot study aimed to estimate effect sizes for future power analysis and to examine the feasibility of the STEAM PBL intervention program for first-year preschool children.

## 2 Methods

### 2.1 Participants

The participants were children from two local kindergartens with distinct curriculum models: Kindergarten A delivered the project-based STEAM curriculum and Kindergarten B adopted the traditional theme-based curriculum. Three first-year preschool classes (3–4-year-olds) were randomly sampled, with two classes following the STEAM curriculum as the experimental group and one class following the traditional curriculum as the control group. One-on-one assessments were administered to all participants.

Given that the test was entirely voluntary for children (i.e., they could choose not to participate or withdraw at any time), and due to occasional absences from preschool over the study period, only 69 children fully completed valid pre-test and post-test assessments (as shown in Table 1). All assessments were voluntary, and parental informed consent was obtained for each participating child.

**Table 1:** Two groups of preschoolers.

Variables	Kindergarten A (*n* = 41)	Kindergarten B (*n* = 28)	t	*p*
Boys	25	10		
Girls	16	18		
Creative potential	16.63 (11.68)	22.39 (13.87)	−1.86^+^	0.067

+, *p* < 0.1.

### 2.2 Measurement instruments

Creative potential is the cognitive ability of individuals to generate novel and useful products or ideas (Runco and Jaeger, 2012). Viewing children’s creative behavior from a future-oriented perspective, this concept has a predictive effect on individuals’ creativity (Silvia et al., 2016).

This study utilized the Thinking Creatively in Action and Movement (TCAM), developed by Torrance in 1981, to evaluate the creative potential of preschool children through the observation of their performance in physical activities and action tasks, as young children primarily express their ideas through actions (Rivera et al., 2020; Torrance, 1981). TCAM is one of the few standardized, age-appropriate, performance-based measures for very young children. For example, Fan et al. (2023) used it to assess the creative potential of children aged 1.5–5 years.

Meanwhile, because the children in this study were entering preschool for the first time, were still adapting to preschool life, and had limited verbal and drawing abilities, a movement-based approach was adopted to help them express their creative ideas and abilities.

The primary scoring dimensions of TCAM consist of four components: fluency, flexibility, originality, and imagination. The assessment comprises four activities:

(1) What is the total number of possibilities? Children were encouraged to use different ways of moving from one location to another, and each method used was recorded.
(2) In what manner can you move? Children were instructed to imitate a tree swaying in the wind, a rabbit running, a fish swimming in water, driving a vehicle, and moving like an elephant, and were evaluated based on their performances.
(3) Are there alternative methods? Children were instructed to place paper cups into a wastebasket using different methods, and each method was recorded.
(4) What purposes may it serve? Children were instructed to describe or demonstrate different uses of a paper cup, and each response was recorded.

Activity 2 assessed imagination, whereas Activities 1, 3, and 4 primarily assessed fluency, originality, and flexibility. Responses were rated on a scale from 0 to 3, while the imagination score utilized a five-point Likert scale, ranging from “no movement” to “simulated very much,” scored from 0 to 5.

The TCAM demonstrated substantial test–retest reliability over a 2-week period, with intraclass correlations ranging from 0.91 to 0.97 (Rivera et al., 2020).

### 2.3 Intervention design

To examine the impact of STEAM project-based curricula on children’s creative potential, this study adopted a quasi-experimental approach, establishing an experimental group (two classes from Kindergarten A) and a control group (one class from Kindergarten B). Kindergarten A implemented a STEAM project inquiry curriculum, following the formative philosophy of high-quality STEAM projects (Peng, 2024).

Two STEAM projects were implemented in the experimental group, one per semester. The first was the STEAM Bionic Project, which aimed to explore how to learn from plants and animals in nature to solve problems in life. The second was the STEAM Bridge Project, which focused on how to prevent bridge fracture or collapse and how to construct impregnable, unique bridges.

Kindergarten B adopted a traditional thematic curriculum, which involved carrying out daily care and group teaching activities in accordance with the regional curriculum syllabus and traditional themes (e.g., themes related to seasonal changes throughout the year and different festivals). The themes implemented in the first semester were I Am in Preschool (Adaptation/September), I Love My Motherland (National Day/October), Colorful Leaves and Fruit (Autumn theme/November), and Winter Festivals (Winter theme/December). The themes implemented in the second semester were Celebrating the New Year (Lantern Festival/February), Spring Is Coming (Spring Equinox and Arbor Day/March), Love My Family (Tomb-Sweeping Day/April), Labor Is Glorious (May Day and Self-care/May), and Happy Children’s Day (I love kindergarten/June).

Below, the STEAM Bionic Project is used as an example to illustrate the core experimental procedures, together with the main differences from the control class. In the experimental group, teachers facilitated STEAM project inquiry using the PP-O model (Peng, 2019; Peng, 2023a) with authentic real-world challenge problems. The PP-O model forms a continuously advancing and internally coherent process that proceeds from phenomenon presentation → problem elicitation and guidance → inquiry through operation.

Therefore, the first and most critical stage involves presenting sufficient, astonishing, or typical phenomena demonstrating nature’s capabilities through multiple approaches. For example, children were introduced to the camouflage abilities of butterflies, dead-leaf butterflies, chameleons, and other animals. Multimodal presentation methods included real objects, models, images, videos, picture books, operating materials, which were provided both in learning centers/corners and in group activities for approximately 1 month.

The teachers’ main roles at this stage were supporter and presenter, rather than remaining at the center of instruction as an instructor, guide, or explainer, as in traditional thematic courses. Teachers then closely observed, listened to, and recorded children’s interests, questions, and concerns during the presentation stage. If the children showed no interest whatsoever, the authentic problem was considered inappropriate for them, and the project did not continue.

The teachers’ main roles during this stage were observer, listener, and recorder, rather than directly teaching children or continuously posing questions to evaluate their answers, as is common in traditional theme-based curricula and many preschool classrooms.

Bottom-up questions from children and top-down analysis conducted by teachers in advance constituted the problem space for this Bionic STEAM project. During the third stage (approximately two additional months), teachers supported children’s hands-on and minds-on inquiry by focusing on one previously identified question at a time.

Each specific activity contained a micro-cycle that ranged from the presentation of typical phenomena focusing on the particular problem (e.g., how to learn from butterflies to hide effectively during a hide-and-seek game, or how to learn from the lotus leaf to design rainproof umbrellas?).

Taking the above as an example, activities included butterfly-finding activities in flower clusters (real or artificial), searching for colorful butterfly cards, dead-leaf butterfly-finding activities in real leaf piles, indoor and outdoor hide-and-seek activities, activities in which children colored, painted, and decorated white shirts before hiding them among various forest scenes, observation of butterfly specimens in the kindergarten museum, butterfly picture-book reading activities, picture-matching activities related to butterflies and their bionic applications in daily life, and small experimental activities exploring how butterflies fly.

The teachers’ main roles during this stage were follower, supporter, listener, and reflector, rather than model, instructor, monitor, leader, or controller.

All of these activities aimed to achieve the core objectives of young children’s STEAM education (Peng, 2023b): stimulating interest in STEAM inquiry (such as interest in paying attention to, exploring, and investigating real-world problems), shaping positive approaches to learning, and promoting STEAM thinking.

### 2.4 Research procedure

The study was conducted in seven phases.

First, consent was obtained from the children’s primary caregivers (before the research), and assent was obtained from the children themselves (before assessment).

Second, three classes (first-year preschoolers) were randomly selected from two preschools. Among these, two classes were assigned to the STEAM PBL experimental group, and the other class served as the traditional theme-based control group.

Third, pre-tests (one-on-one assessments of creative potential) were conducted for children in both groups within the first 1–2 weeks after enrollment.

Fourth, the experimental group implemented a 1-year intervention (with ongoing support from the researcher, including training, communication, and monitoring), while the control group followed the regular and traditional curriculum over the same period.

Fifth, after one academic year of curriculum implementation, post-tests (identical to the pre-tests) were conducted.

Data from children who were absent or withdrew were excluded, yielding complete data from 69 children (experimental: n = 41; control: n = 28).

Sixth, the researcher watched the assessment videos, analyzed each child’s performance across the various dimensions of creative potential, and examined inter-rater reliability.

Finally, the study analyzed differences in creative potential between the two groups and their respective longitudinal changes.

### 2.5 Statistical analysis

SPSS 29.0 was used to conduct statistical analyses of the horizontal differences and vertical changes in creative potential between the two groups of children before and after the implementation of the curriculum.

## 3 Results

### 3.1 The basic features of 3–4-year-old children’s creative potential

A holistic descriptive statistical analysis was conducted on the pre-test scores of creative potential among kindergarten children who participated in the assessment. As shown in Table 2, children’s overall creative potential conformed to a normal distribution, with a total mean score of 18.97 and a standard deviation (SD) of 12.84. The relatively large SD indicated a high degree of data dispersion, which reflected obvious individual differences in creative potential among these children. In terms of the mean scores across sub-dimensions, the score for flexibility was the lowest, while the scores of the other three dimensions were comparable.

**Table 2:** Descriptive statistics of children’s creative potential.

Variables	*N*	Min	Max	Mean	SD
Fluency	69	0	23	4.94	4.70
Flexibility	69	0	8	2.67	1.95
Originality	69	0	34	6.22	6.68
Imagination	69	2	8	5.15	1.30
Creative Potential	69	2	61	18.97	12.84

SD, standard deviation; Min, minimum; Max, maximum.

In terms of the overall changes observed 1 year later, the total scores of creative potential and the scores across all sub-dimensions of the assessed children at Time 2 (T2) were all higher than those at Time 1 (T1), with the only exception being the imagination dimension, for which scores showed a decrease (imagination: M₁ = 5.15 at T1, M₂ = 4.71 at T2).

In addition, an analysis of the extreme values and SDs at T1 and T2 revealed that the minimum scores of creative potential and its sub-dimensions remained identical across the two time points, whereas the maximum scores and SDs of all sub-dimensions at T2 were higher than those at T1.

A difference test conducted on the SDs between the two time points indicated a marginally significant difference (*t* = −2.402, *p* = 0.074 < 0.1). These results demonstrated that individual differences in children’s creative potential exhibited a tendency to expand.

### 3.2 Between-group comparison of children’s creative potential at pre-test and post-test

First, a comparison was conducted between the pre-test scores of creative potential of children in the experimental group and those of children in the control group. As shown in Table 3, the mean scores of fluency, flexibility, originality, imagination, and total creative potential among children in the control group were all higher than those in the experimental group.

**Table 3:** Comparative analysis of pre-test results for children’s creative potential.

Variables	Experimental group		Control group		*t*	*p*
	*N*	M ± SD	*N*	M ± SD		
Fluency	41	4.15 ± 4.39	28	6.11 ± 4.97	−1.73^+^	0.089
Flexibility	41	2.51 ± 2.04	28	2.89 ± 1.83	−0.79	0.431
Originality	41	4.98 ± 5.59	28	8.04 ± 7.76	−1.91^+^	0.061
Imagination	41	5.00 ± 1.20	28	5.36 ± 1.42	−1.13	0.265
Creative Potential	41	16.63 ± 11.68	28	22.39 ± 13.67	−1.86^+^	0.067

M, mean; SD, standard deviation. +, *p* < 0.1.

Independent-samples *t*-tests revealed that such differences reached marginal significance for total creative potential as well as the fluency and originality dimensions (*p* < 0.1). Specifically, the control group scored higher than the experimental group in total creative potential and the sub-dimensions of fluency and originality, whereas no significant differences were found in the other dimensions.

This indicated that before curriculum intervention, there were certain initial differences in the developmental level of creative potential between the two groups, with children in the control group demonstrating slightly better overall performance in creative potential than those in the experimental group.

A further comparison was conducted on the post-test scores of creative potential among children from different groups. The results indicated that the experimental group scored higher than the control group in terms of total creative potential and all its sub-dimensions.

Independent-samples *t*-tests showed that the differences in total creative potential and most sub-dimensions between the two groups were not statistically significant. Nevertheless, the between-group difference in the flexibility dimension reached marginal significance (*t* = 1.91, *p* < 0.10), suggesting a tentative positive tendency for the experimental group to show a relatively higher level of flexibility than the control group.

Taken together, the above results revealed that the control group exhibited better performance in creative potential than the experimental group at the pre-test stage, yet 1 year later, children in the experimental group had comprehensively surpassed their counterparts in the control group in overall creative potential.

To account for pre-test inter-individual differences, analysis of covariance (ANCOVA) was conducted with pre-test scores as the covariate and group as the fixed factor. After controlling for baseline performance, the experimental group showed higher adjusted post-test means than the control group across all subscales of creative potential, although the group differences did not reach statistical significance.

The specific results were as follows: fluency: *F* (1, 66) = 0.21, *p* = 0.649, ηp^2^ = 0.0003; flexibility: *F* (1, 66) = 2.78, *p* = 0.100, ηp^2^ = 0.040; originality: *F* (1, 66) = 0.85, *p* = 0.361, ηp^2^ = 0.013; imagination: *F* (1, 66) = 0.55, *p* = 0.461, ηp^2^ = 0.008; total creative potential: *F* (1, 66) = 1.02, *p* = 0.316, ηp^2^ = 0.015.

Effect sizes (Cohen’s d) ranged from 0.47 to 0.58, indicating moderate effects.

### 3.3 Between-group comparison of longitudinal changes in children’s creative potential

To examine whether each group demonstrated significant longitudinal changes in creative potential after 1 year, paired-samples *t*-tests were conducted for the experimental group and the control group separately (Table 4).

**Table 4:** Changes in children’s creative potential under different treatments.

Variables	Experimental group (*N* = 34)		t	*p*	Control group (*N* = 27)		t	*p*
	T1	T2			T1	T2		
Fluency	3.97 ± 4.43	7.88 ± 10.68	−1.98^+^	0.056	6.25 ± 5.00	6.19 ± 11.06	0.04	0.973
Flexibility	2.47 ± 2.00	4.15 ± 2.95	−3.04^**^	0.005	2.93 ± 1.86	2.96 ± 3.30	−0.07	0.948
Originality	4.35 ± 4.74	9.32 ± 14.74	−1.92^+^	0.064	8.26 ± 7.82	4.81 ± 9.33	1.58	0.127
Imagination	5.12 ± 1.17	4.85 ± 1.60	0.79	0.436	4.93 ± 1.21	4.52 ± 1.48	1.19	0.245
Creative potential	15.91 ± 11.01	26.21 ± 28.14	−2.03^+^	0.051	22.37 ± 13.67	18.48 ± 21.20	0.92	0.365

Thirty-four children had complete data on all dimensions in the experimental group, and 27 children had complete data in the control group. +, *p* < 01; **, *p* < 01.

The results indicated that, except for imagination, the mean scores and SDs of fluency, flexibility, originality, and creative potential in the experimental group were higher at T2 than at T1. The paired-samples *t*-test indicated that all dimensions of the experimental group, except for imagination, achieved significant or marginal significance (fluency: *t* = −1.98, *p* = 0.056 < 0.1; flexibility: *t* = −3.04, *p* = 0.005 < 0.01; originality: *t* = −1.92, *p* = 0.064 < 0.1; creative potential: *t* = −2.03, *p* = 0.051 < 0.1).

The disparities between the two time points in the control group were not statistically significant. Except for flexibility, the mean values for all dimensions were higher at T1 than at T2, whereas the SDs were higher at T2 than at T1. This indicates that the creative potential of children in the experimental group progressively improved over time, with individual differences continuing to widen, whereas the control group exhibited expanding individual differences without an increase in creative potential.

**Figure 1:**
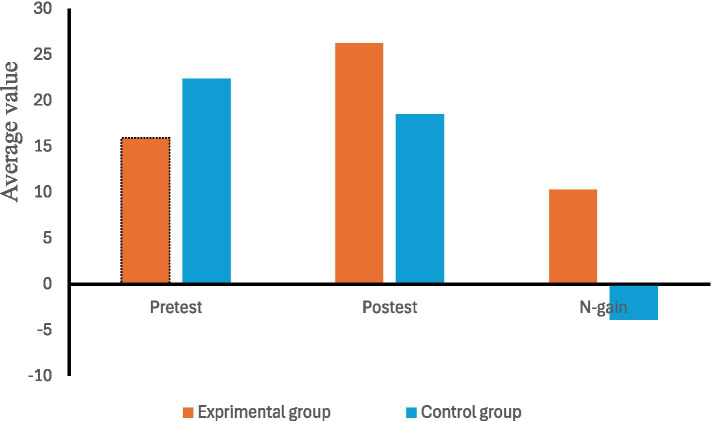
Comparison of creative potential scores between the two groups.

Figure 1 illustrates the pre-test, post-test, and N-Gain scores for creative potential in both groups. Consistent with the statistical results reported above, the figure shows that the experimental group exhibited a positive N-Gain, whereas the control group showed little to no improvement.

To further examine whether the longitudinal changes differed significantly between the two groups, a 2 (group: experimental vs. control) × 2 (time: pre-test vs. post-test) repeated-measures analysis of variance (ANOVA) was performed for each variable.

For creative potential (total score), the Group × Time interaction was significant, *F* (1, 67) = 5.21, *p* = 0.025, ηp^2^ = 0.072. Simple-effects analysis further revealed that the experimental group improved significantly more over time (pre-test to post-test) in creative potential (mean difference = 10.30, standard error [SE] = 4.72, *t* (40) = 2.18, *p* = 0.035, Cohen’s d = 0.34), whereas the control group did not show a significant change (mean difference = −3.91, SE = 4.76, t (27) = −0.82, *p* = 0.419).

No other significant interactions were observed for the remaining variables.

## 4 Discussion

### 4.1 Project-based STEAM curriculum has a moderate effect on fostering the creative potential of 3–4-year-old children

The effects of STEAM education programs have attracted increasing research attention in recent years. A meta-analysis conducted in Korea reported an overall medium effect size (Jeong et al., 2023). In terms of school level, the effect size ranked as follows: elementary school > middle school > high school. However, whether such effects extend to creative potential at the preschool level, especially in the first preschool year, remains unclear. Creative potential refers to the latent cognitive resources and individual predispositions that can be transformed into publicly recognized creative achievements under appropriate conditions. Existing studies have found that STEAM curricula, especially those integrating PBL and arts, can significantly enhance children’s creative thinking, encompassing fluency, flexibility, and originality (Aguilera and Ortiz-Revilla, 2021; Chang et al., 2023; Kuo, 2024; Lage-Gómez and Ros, 2024; Lu et al., 2022; Ozkan and Topsakal, 2019; Putri et al., 2023). Some studies have also suggested that the promotional impact of conventional theme-based curricula on creative potential may be limited (Saputra et al., 2023).

The present study further demonstrated that project-based STEAM curricula possess certain advantages compared with traditional thematic curricula in promoting the creative potential of 3–4-year-old children. Pre-test comparisons indicated that the control group had a higher baseline level than the experimental group in the total score of creative potential and multiple dimensions. After 1 year of intervention, the post-test results showed that the mean scores of the experimental group in total creative potential and all its dimensions surpassed those of the control group, with a marginally significant group difference observed in the dimension of flexibility. These results indicated that the distinct initial advantage of the control group had disappeared. The control group showed no sustained development in creative potential, whereas the experimental group exhibited a growth trend. ANCOVA results indicated moderate effect sizes (Cohen’s d = 0.47–0.58), suggesting practically meaningful differences between groups despite the lack of statistical significance given the pilot study’s limited sample size. These findings provide preliminary empirical support for the potential benefits of the project-based STEAM curriculum for 3–4-year-olds’ creative potential.

A further within-group longitudinal comparison of changes supported these findings. For instance, in the experimental group, post-test scores (T2) were higher than pre-test scores (T1) in total creative potential and in the dimensions of fluency, flexibility, and originality, except for imagination. All these changes reached statistical or marginal significance (see Table 4). In contrast, in the control group, the pre-test scores (T1) were higher than the post-test scores (T2) in total creative potential and most dimensions, indicating a decline rather than an improvement in creative potential. Further statistical analysis based on the 2 (group) × 2 (time) repeated-measures ANOVA also indicated that the experimental group showed significant improvements over time, whereas the control group exhibited no significant changes. These results demonstrated that the STEAM project-based curriculum enabled young children in the experimental group to shift from a baseline disadvantage to an apparent developmental advantage in creative potential, reflecting the positive effects of the STEAM project-based curriculum on young children’s creative potential relative to traditional thematic curricula.

The findings of Zahra et al. (2023) and Lu et al. (2022) indicate that STEAM courses can markedly enhance cognitive creativity in terms of flexibility and originality; however, their studies lacked a control group, preventing them from isolating the course’s effects from those of general PBL. This study, by incorporating a control group, suggested that STEAM courses may enhance creative potential and illustrated potential advantages over thematic curricula within the preschool population. These advantages may derive from the application of the PP-O model (Peng, 2019; Peng, 2023a) in authentic project-based STEAM inquiry, in which teachers primarily serve as supporters and undertake substantial preparatory work behind the scenes to better follow children’s interests and self-generated questions, rather than leading or dominating children’s exploration. STEAM curricula offer greater opportunities for independent exploration and expression (Aguilera and Ortiz-Revilla, 2021; Kuo, 2024).

Traditional curricula predominantly focus on teacher-directed knowledge dissemination, prioritizing standardized responses and uniform procedures, while failing to facilitate children’s autonomous exploration and creative expression (Cheng et al., 2022; Gracia et al., 2024; Ozkan and Topsakal, 2019). This paradigm effectively reinforces children’s cognitive processes and inadequately addresses the open and exploratory learning requirements essential for fostering creative potential.

### 4.2 The promotion effect of the project-based STEAM curriculum is dimension-specific

While the project-based STEAM curriculum positively influences overall creative potential, its promotional effects are unbalanced and exhibit clear dimensional specificity. This is particularly apparent in the change patterns of each dimension within the experimental group. The curriculum is more effective in enhancing cognitive flexibility (the capacity to shift between different perspectives or problem-solving strategies in response to changing task demands, *p* < 0.01), followed by originality (the ability to produce innovative ideas, *p* < 0.1) and fluency (the quantity of ideas generated, *p* < 0.1), whereas its impact on imagination (the capacity to create mental imagery, *p* > 0.1) is comparatively restricted.

STEAM PBL prioritizes interdisciplinary integration and practical problem-solving, necessitating learners to regularly engage in cognitive transformation and reorganization. It directs children to engage in extensive exploration of a complex issue over several weeks or more, as opposed to rapid brainstorming sessions designed to produce a multitude of ideas (Aziz and Abu Bakar, 2021). Consequently, flexibility—the ability to shift between different cognitive approaches and solution strategies—was explicitly practiced, and marginal gains in originality and fluency were also observed. The gains in originality and fluency, though marginal, are consistent with the structured yet open-ended nature of STEAM inquiry, which requires children to generate multiple solutions to authentic problems while encouraging novel approaches (Erol et al., 2023; Habibi, 2023; Newton, 2001).

However, the enhancement of imagination was limited. This pattern is evident in the changes in the extreme values within the score distribution (refer to Table 4). The experimental group’s highest score in the originality dimension increased from 24 to 73, significantly surpassing the control group’s score increase from 34 to 41; regarding creative potential, its upper limit increased from 60 to 153. Conversely, in the imagination dimension, while the maximum value of the experimental group showed a modest increase (8 → 10), the mean and intra-group differences remained largely unchanged. The limited enhancement of imagination requires consideration of both measurement issues and developmental appropriateness. Previous research has noted no significant development of imagination in 3–4-year-old children (Miao et al., 2010), and the present study similarly found no significant intervention effects on this dimension. Imagination may be the most stable and difficult-to-cultivate aspect of creative potential at this age, perhaps because its development depends on different mechanisms than those targeted by STEAM PBL. Unlike the cognitive processes directly engaged by interdisciplinary exploration and problem-solving (Aguilera and Ortiz-Revilla, 2021; Atikah and Biru, 2024; Chang et al., 2023; Lage-Gómez and Ros, 2024; Wilson et al., 2021), imaginative capacity may require a more prolonged and immersive setting than cognitive skills (Lu et al., 2022). As imagination is a unique ability of humans (Chen, 2013) that cannot be replaced by AI technology, and as it may be increasingly important in the future (Reeves and Fuller, 2020), this dimension merits further exploration in future studies and heightened attention in educational practice.

Taken together, future studies may further explore the underlying mechanisms through which teachers’ concrete scaffolding behaviors, project structure and activity model, and support for children’s engagement shape specific dimensions or overall creative potential development. Teachers may serve as important mediators linking curriculum design and creativity-related outcomes (Peng, 2024). Unpacking the internal characteristics of project-based STEAM curricula can help identify their most effective features, which carry important implications for curriculum optimization in early childhood education.

### 4.3 Individual differences and the catalytic effect of the project-based STEAM curriculum

All tested children exhibited significant individual differences across all dimensions of creative potential, as evidenced by the larger SDs in the post-test (T2) compared to the pre-test (T1). For instance, the SD for fluency increased from 4.70 to 9.23, while the SD for uniqueness rose from 6.68 to 11.82. These individual differences increased over time, particularly in the total score of creative potential, with its variability (SD) rising dramatically from 12.84 to 23.17. This indicated a notable widening trend in such individual differences over time.

Analysis of this overarching trend reveals that STEAM project curricula and regular academic curricula exert distinct effects. The data in Table 4 indicate a notable increase in the SDs of the experimental group (project-based STEAM curriculum) in the post-test dimensions, as illustrated by the fluency SD rising from 4.43 to 10.68 and the creative potential SD increasing from 11.01 to 28.14. The intra-group and inter-group analyses of the difference between the pre-test and post-test indicated that the experimental group exhibited considerably greater improvement in flexibility, originality, and overall creative potential compared to the control group (*p* < 0.05). Conversely, the SDs of the control group (traditional theme-based curriculum) in each dimension exhibited minimal variation, with the difference between the pre-test and post-test largely being nonsignificant (*p* > 0.05).

This indicates that the open, autonomous, and diverse expressive environment of STEAM curricula is likely to yield varied promotional effects due to differing initial cognitive preparedness, learning styles, and interest orientations among young children, thereby intensifying individual differences in abilities within the group (Aguilera and Ortiz-Revilla, 2021; Conradty and Bogner, 2020; Conradty et al., 2020; Zhan et al., 2022). Children exhibiting greater creative potential or alignment with the curriculum design—such as those possessing diverse interests or distinctive hobbies—may derive enhanced benefits from it, resulting in the swift realization of their potential. Conversely, the traditional theme-based curriculum may play a relatively constrained role in fostering individual differences due to its more uniform structure, and it may not significantly stimulate the potential of certain children, particularly those with a high baseline, as effectively as the STEAM curriculum.

The observed increase in individual variability in this study presents a common difficulty in creative potential intervention research: the average effect may obscure intricate and diversified response patterns. The inter-group effect of the experimental group did not attain a statistically significant level in certain categories. Besides the constraint of sample size, this finding may also stem from the considerable variety of individual response curves; the substantial advancement of certain children was offset by the slower development of others.

### 4.4 Limitations

Although this research represents a significant advancement in education by addressing critical gaps in the literature and attempts to establish a research foundation for developing and implementing STEAM PBL at the beginning of preschool years, it has several limitations closely related to its participants and research procedures.

First, the sample scope was limited: only 69 children completed both pre- and post-tests by the end of the study because of frequent absences. This might reduce statistical power and representativeness. Nevertheless, this pilot study may provide preliminary evidence and lay the foundation for subsequent large-scale research. For example, future research may adopt an optimized sample size to improve statistical power, enrich the comparison conditions, and include more diverse samples based on the feasibility and preliminary effects verified in this pilot work. In this way, the current study serves as a necessary preliminary phase and provides clear guidance for subsequent large-scale empirical studies.

Second, this was a quasi-experimental design, where the experimental group and control group were sampled from two kindergartens with distinct curricula, leading to potential selection bias from confounding factors (e.g., teacher quality) that could not be fully controlled.

Third, the study did not explore the specific mechanisms through which the STEAM curriculum affected children’s creative potential. Future research might utilize larger sample sizes and implement person-centered analytical approaches with the use of multiple informants and measures to assess creative potential in preschool children (Cieslik et al., 2026), such as latent class analysis, to ascertain which subgroups of children derive the greatest benefit from STEAM projects, thereby furnishing empirical evidence for targeted and personalized educational interventions.

## Data Availability

The raw data supporting the conclusions of this article will be made available by the authors, without undue reservation.
